# Parallel G-quadruplex Structures Increase Cellular Uptake and Cytotoxicity of 5-Fluoro-2′-deoxyuridine Oligomers in 5-Fluorouracil Resistant Cells

**DOI:** 10.3390/molecules26061741

**Published:** 2021-03-20

**Authors:** Anna Clua, Carme Fàbrega, Jesús García-Chica, Santiago Grijalvo, Ramon Eritja

**Affiliations:** 1 Institute for Advanced Chemistry of Catalonia (IQAC-CSIC), ) Jordi Girona 18-26, E-08034 Barcelona, Spain; anna.clua@iqac.csic.es (A.C.); cgcnqb@cid.csic.es (C.F.); garciachicajesus@gmail.com (J.G.-C.); sgrgma@cid.csic.es (S.G.); 2 Networking Center on Bioengineering, Biomaterials and Nanomedicine (CIBER-BBN), Jordi Girona 18-26, E-08034 Barcelona, Spain

**Keywords:** cellular uptake, drug delivery, 5-fluoro-2′-deoxyuridine, G-quadruplex, nanocarriers, nanostructures, drug resistance, cancer therapy, apoptosis, oligonucleotide prodrugs

## Abstract

Fluoropyrimidines, such as 5-fluorouracil (5-FU) and related prodrugs have been considered first-line chemotherapy agents for the treatment of colorectal cancer. However, poor specificity and tumor cell resistance remain major limiting bottlenecks. G-quadruplexes, have been suggested as preferred nanostructures for enhancing cellular uptake mediated by G-quadruplex binding proteins which are abundant at the membranes of some tumor cells. In the current study, we propose a new strategy to deliver 5-fluoro-2′-deoxyuridine (5-FdU) monophosphate, the main active drug from 5-FU derivatives that may circumvent the cellular mechanisms of FU-resistant cancer cells. Two G-quadruplexes delivery systems containing four and six G-tetrads ((TG_4_T) and (TG_6_T)) linked to a FdU oligonucleotide were synthesized. Biophysical studies show that the G-quadruplex parallel structures are not affected by the incorporation of the 5 units of FdU at the 5’-end. Internalization studies confirmed the ability of such G-quadruplex nanostructures to facilitate the transport of the FdU pentamer and increase its cytotoxic effect relative to conventional FU drug in FU-resistant colorectal cancer cells. These results suggest that FdU oligomers linked to G-quadruplex parallel sequences may be a promising strategy to deliver fluoropyrimidines to cancer cells.

## 1. Introduction

Cancer is currently one of the world’s leading causes of death, with over 18.1 million cases and 9.6 million deaths in 2018 [1]. Cancer is known to be a myriad of different diseases with high variability from tissue to tissue; as well as from patient to patient and even between the same types of cancer. This variability makes it difficult to find a unique treatment that fits all types of cancer [2].

5-Fluorouracil (5-FU) is one of the most successful drugs used in chemotherapy for the treatment of diverse severe cancers, including colorectal ones. 5-FU is an equivalent of uracil (U) in which the hydrogen atom at the C-5 position is replaced by a fluorine atom. This structural similarity enables a quick entrance into the cells by the uracil transport mechanism [3]. The antitumoral activity of 5-FU is produced after its intracellular conversion to 5-fluoro-2′-deoxyuridine monophosphate (FdUMP), 5-fluoro- 2′-deoxyuridine triphosphate (FdUTP) and 5-fluorouridine triphosphate (FUTP). These three active metabolites interfere in multiple critical cellular processes. For instance, FdUMP binds covalently to thymidylate synthase (TS) and forms a stable ternary complex with a reduced folate [4,5,6]. As a consequence, thymidine monophosphate (dTMP) synthesis is inhibited provoking “thymineless cell death” [7,8]. Whereas, FdUTP is misintegrated to DNA producing a frequency increase of base excision repair (BER) processes, which ends in DNA strands break and cell death [9]. Finally, FUTP is incorporated in RNA synthesis instead of uracil, leading to the damage of the normal RNA processing and function [10,11,12].

One of the major problems described in clinical practice is 5-FU cell resistance. Cancer cells can become immune to this drug mainly by increasing 5-FU catabolism by dihydropyrimidine dehydrogenase (DPD) [13,14] or by scaling the rate of dTMP biosynthesis [15]. Moreover, other mechanisms of resistance are recognized to take part in this loss of activity [16]. As a consequence of all these factors, 5-FU bioavailability is largely reduced so, the 5-FU dose has to be increased to continue seeing a clinical effect. Furthermore, the lack of specificity for cancer cells provokes a large number of side effects, as dermatological, hematological, neural and cardiac dysfunctions, besides toxicity on the gastrointestinal tract [17,18,19]. Seeing that, the delivery and targeting of 5-FU have room for improvements. Over the past years, many attempts have been made to synthesize more effective and active fluoropyrimidine drug analogs by oral administration, such as Ftorafur (FTO, 1-(2-tetrahydrofuryl)-5-fluorouracil, Tegafur or Futraful) and 5′-deoxy-5-fluorouridine (5′d5-FUrd, doxifluridine or Furtulon) [20]. Another example includes the enzymatically activated prodrug, capecitabine [21,22], tegafur-uracil [23] and S-1 [24,25]. Such prodrugs are based on the metabolic conversion to 5-FU as their primary mechanism of cytotoxicity. Another strategy used to improve 5-FU efficiency is its combination with other active compounds, such as folinic acid, irinotecan, leucovorin and oxaliplatin, in order to enhance a synergistic anticancer response [26,27,28].

Nowadays, the aforementioned 5-FU analogs are employed clinically. Having said that, the essential need for novel drug delivery systems capable of reducing cytotoxicity by avoiding healthy cells, increasing drug bioavailability and enhancing drug release into target cells is known [13,14,29]. Several viral vectors such as adenoviruses or retroviruses have been used in clinical trials presenting high transfection efficiencies [30]. However, some concerns regarding immunogenicity or recombination of oncogenes must be overcome. In contrast, non-viral vectors such as aptamers [31], cell-penetrating peptides [32], gold nanoparticles [33], lipids [34], niosomes [35,36], or polymers [37] have come up as promising alternatives to deliver nucleic acids safely. Despite all this progress done in the search for new formulations for nucleic acids, there is a need for novel and more efficient delivery systems.

In the last decades, aptamers have proved to be very attractive as therapeutic agents mainly for their enhanced cellular uptake, lower manufacture costs and non-immunogenicity [38]. Diverse aptamers have been discovered and evaluated against different human diseases, such as age-related macular degeneration [39], diabetes [40], inflammation [41], neurodegenerative diseases [42], thrombosis [43], etc. Many bioactive aptamers are G-rich oligonucleotides sharing a non-canonical nucleic acid structure. The core unit is composed of guanines that interact between them through Hoogsteen base pairing, forming planar arrangements of four of them interacting (G-tetrads). The stacking of two or more guanine tetrads generates a G-quadruplex motif, which is further stabilized by monovalent cations (in particular K^+^ and Na^+^) hosted in the central cavity of the G-quadruplex [44]. Depending on the number of contiguous G-tracts and the loop size, these sequences can adopt various G-quadruplex topologies, which are normally classified as parallel-, antiparallel- and hybrid-type parallel-antiparallel-stranded conformations [45,46]. In addition, they can be unimolecular (monomeric), bimolecular (dimeric) and tetramolecular (tetrameric) [46].

G-quadruplexes have a very stable structure under physiological conditions [47] and they are widely present in the genome [48]. Furthermore, several studies provide in vivo evidence of its significant roles in biological processes, such as chromosome maintenance, telomerase dysfunction and regulation of several oncogenes expression [49,50,51,52]. Finally, G-quadruplexes recognized selectively very different protein targets such as Stat3 [53] or nucleolin [54] in cancer cells. The high affinity and specific recognition for specific targets, similar to antibodies, make aptamers very helpful for targeting and drug delivery. Indeed, few aptamers have been reported to have per se antiproliferative effects against colon cancer [55] and ErbB2-positive breast cancer [56]. In addition, aptamers such as AS1411 are capable to form G-quadruplex and enhance cellular uptake of several drugs or nanomaterials mediated by G-quadruplex binding proteins which are abundant at the membranes of some tumoral cells [57,58,59]. Recently parallel quadruplexes carrying lipids or positively-charged amino acids have been used as antivirals [60] and as cellular uptake enhancers for antisense oligonucleotides [61,62].Oligonucleotides made from several units of antiproliferative nucleosides have gained particular interest as prodrugs [63]. These oligomers are intracellularly cleaved by nucleases generating nucleoside monophosphates that are the active form of these antiproliferative nucleosides [64]. Recently this strategy has been successfully used for the treatment of colon cancer metastasis in mice using a nanoprotein assembly carrying FdU pentanucleotide and the T22 peptide with high affinity to CXCR_4_ receptor abundant in metastatic cells [65]. Moreover, floxuridine oligomers have been used to create DNA nanoarchitectures with antiproliferative properties [66,67].

In the present work, we inspected the ability of parallel G-quadruplexes to deliver floxuridine oligonucleotides into different types of cancer cells. To pursue this goal, two TG_n_T (n = 4 and 6) G-quadruplexes were synthesized and compared in terms of structural stability, cell uptake and cytotoxic effects. Firstly, oligonucleotides composed of five or ten molecules of 5-fluoro-2′-deoxyuridine (floxuridine, FdU) were inserted in the 5′-end of inherent TG_n_T, to act as therapeutic agents. Once the G-quadruplexes were formed the biophysical properties of these nanoconjugates have been tested. Finally, the internalization ability and the antiproliferative action of these oligoFdU-G-quadruplexes have been evaluated in FU-resistant cell lines.

## 2. Results and Discussion

### 2.1. Synthesis and Characterization of the G-quadruplexes Nanostructures

To investigate the suitability of G-quadruplexes nanostructures as delivery systems forFdU oligomers, we selected two short single-strand sequences TGGGGT [(TG_4_T)] and TGGGGGGT [(TG_6_T)] capable of self-assemble in a parallel G-quadruplex by a simple annealing process. At these same structures, a pentamer of FdUs (FdU_5_) was incorporated to the 5′-end in order to see if this modification affects the G-quadruplex formation. 

To this end, we have prepared (FdU)_5_-TGGGGT and (FdU)_5_-TGGGGGGT oligonucleotides and the corresponding control oligonucleotides (FdU)_5_-T_8_ as well as their corresponding fluorescein-(FAM)-labeled oligonucleotides. All the oligonucleotides were prepared by solid-phase phosphoramidite protocols. Sequences and mass spectrometry characterization data are shown in Table 1.

**Figure 1:**
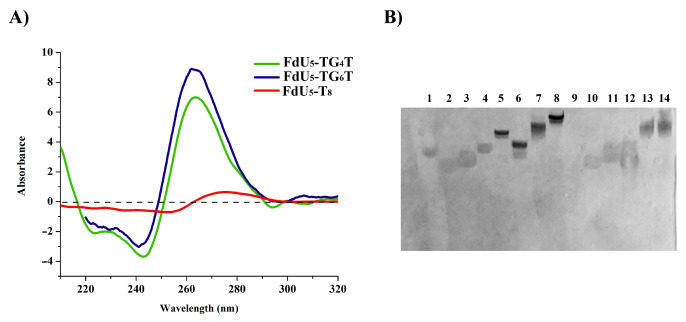
Characterization of the G-quadruplex structures: (**A**) Circular dichroism oligonucleotides in phosphate-buffered saline (PBS) buffer pH 7.4. (**B**) 20% Polyacrylamide gel electrophoresis analysis of the structures formed upon incubation of the different DNA sequences in TBE buffer supplemented with 100 mMKCl. Lanes (1) dye; (2) T_8_; (3) T_12_; (4) TG_4_T; (5) FdU_5_-TG_4_T; (6) TG_6_T; (7) FdU_5_-TG_6_T; (8) FdU_10_-TG_6_T; (9) empty lane; (10) Fam-T_8_; (11) Fam-T_12_; (12) Fam-FdU_5_-T_8_; (13) Fam-FdU_5_-TG_4_T; (14) Fam-FdU_5_-TG_6_T. The gel was stained with Stains-All.

The formation of the G-quadruplex nanostructures was confirmed by circular dichroism (CD) and electrophoretic mobility. CD spectra of floxuridine-modified G-quadruplexes are shown in Figure 1A. Both floxuridine-TG_4_T and floxuridine TG_6_T display the characteristic parallel-stranded tetramolecular G-quadruplex structure at 20 °C with a negative and a positive band at 240 nm and 260 nm respectively. In contrast, the CD spectrum of the control oligonucleotide floxuridine-T_8_ did not have any of the G-quadruplex bands (Figure 1A). The CD spectra of the two unmodified G-quadruplexes (TG_4_T and TG_6_T) [61,62]. had the same CD spectra than the corresponding floxuridine sequences as expected (data not shown).

To confirm these results the FdU-modified G-quadruplexes were studied by 20% native polyacrylamide gel electrophoresis (PAGE) in the presence of 100 mM KCl. The FdU-modified nanostructures exhibited a single band with clear reduced mobility similar to the G-quadruplex controls (TG_4_T and TG_6_T) in gel electrophoresis (Figure 1B and Figure S1). The extra FdU nucleotides produce a small less shift in mobility. As expected the T_8_ and T_12_ control sequence run as a single band with faster mobility in the gel. On the other, the presence of a fluorescein molecule produces retardation in the gel mobility in all the sequences without altering apparently the G-quadruplexes structure as anticipated. We have also observed that We have also observed that the samples that form G-quadruplex presented a stronger stain with Stains-All. These results indicated that the introduction of five FdU molecules and/or the fluorescein fluorophore pendant at the 5′-end of the oligonucleotides did not affect significantly the parallel G-quadruplex structure.

### 2.2. Internalization Effect of FdU-Modified G-quadruplexes Nanostructures

Three different cancer cell lines: HeLa, HTB-38 and HCC2998 were selected. We aimed to evaluate the potential effect of the G-quadruplex structure to enhance the cellular uptake of FdU oligomers by G-quadruplex protein-recognition described for some receptors present in cancer cells [38,68]. Secondly, we selected two colorectal cancer cells due to their different levels of sensitivity to 5-FU [69]. To perform the study of the cell uptake of the G-quadruplex structures by flow cytometry, the oligonucleotides responsible to form the nanostructures were labeled with the fluorescein phosphoramidite (FAM) at the 5′-end (Table 1). This fluorescent dye was used according to experimental requirements in terms of synthesis, purification and subsequent formation of the G-quadruplex.

A major concern was the quenching of the membrane-bound species in order to only quantify the internalization of the G-quadruplex. The most common way to quench membrane-bound fluorescein-labeled compounds for flow cytometry analyses is the use of Trypan Blue [70].

To this aim, we performed a preliminary internalization experiment of the control G-quadruplexes internalization by treating the cells with and without Trypan Blue to quench the extracellular fluoresceine and to measure only the intracellular fluoresceine signal. In both experiments, we observed the same internalization fluorescence indicating that all the G-quadruplexes were completely internalized (data not shown). These results allowed us to perform all the internalization experiments without the presence of trypan blue. 

**Figure 2:**
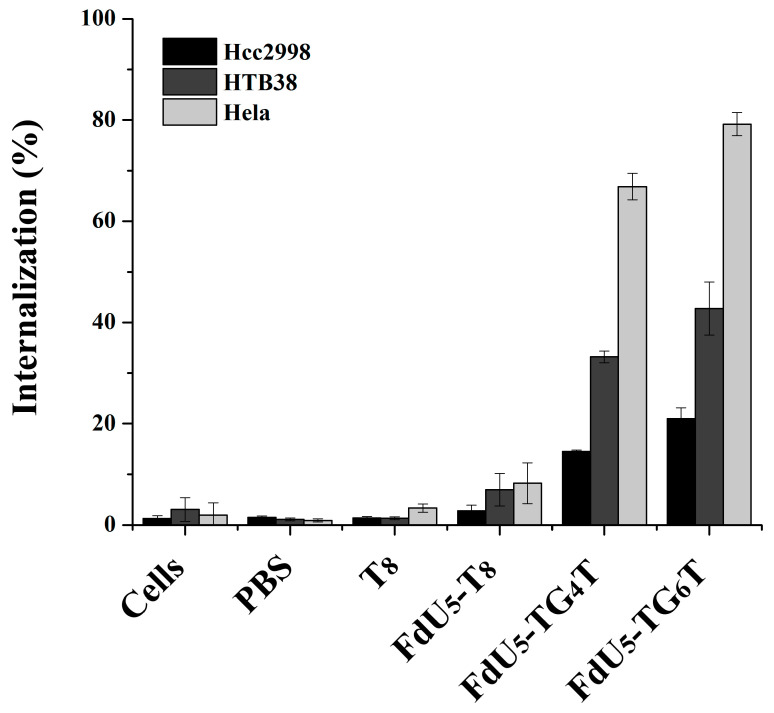
Intracellular uptake of G-quadruplex nanostructures HeLa, and two colorectal cancer cells (HCC2998 and HTB38). Cells were incubated separately with the native and modified G-quadruplexes at 1 µM concentration, and the internalization is shown in the bar graphs HCC2998 (black), HTB38 (dark grey) and HeLa (light grey). Error bars represent the standard deviation (SD) of two independent experiments in duplicate.

Figure 2 shows cell uptake for all different nanostructures with FdU and T_8_ control per each cancer line. The internalization effect of the TG_4_T and TG_6_T controls are shown in Figure S2. It can be observed that (TG_4_T) and (TG_6_T) internalize to a larger extent compared with T_8_ strand control, not able to form G-tetrads. The internalization is higher in (FdU)_5_-TG_6_T than in (FdU)_5_-TG_4_T in all studied cells, suggesting a possible positive effect due to the presence of the extra two quartets in the nanostructure stability making easier its recognition by the cellular receptors. Moreover the cellular uptake is different in each cell line, having the maximum internalization of 80% in HeLa cells. These results are not surprising if we take into account that HeLa cells have a large number of nucleolin receptors [71,72]. The internalization effect of the two G-quadruplexes is larger in HTB38 cells than in HCC2998 cells implicating a potential difference in the number of G-quadruplex receptors between these two colorectal cancer cell lines.

### 2.3. Antiproliferative Effect of FdU-Modified G-quadruplexes Nanostructures

To evaluate the cytotoxic activity induced by the G-quadruplexes (FdU)_5_ we have performed MTT (3-(4,5-dimethylthiazol-2-yl)-2,5-diphenyltetrazolium bromide) and apoptosis assays. The MTT experiments were done to evaluate the cytotoxic effect while apoptosis assays were conducted to compare the cell death of the two G-quadruplex nanostructures and to identify the mechanism involved in the reduction of cell viability. 

Firstly, MTT assays were conducted with the two G-quadruplex motifs without the pro-drug attached and the T_12_ control sequence, which hasn’t influence in the cell proliferation process. These results imply that these nanostructures can be used as nanocarriers of FdU oligomers (Figure S3). Secondly, we studied the cytotoxic response of these cell lines facing the reference anticancer drugs, 5-FU and 5-fluoro-2′-deoxyuridine (FdU), at concentrations ranging from 10 nM to 1 µM. Comprising, by this way, the values of FdU included in DNA nanostructures and those of 5-FU applied clinically [73] (Figure S4). FdU was selected as a control drug since it is considered more efficient in vitro than 5-FU. However, undergoes rapid degradation in vivo into the nucleobase 5-FU being non-advantageous in the clinical practice [73]. Comparing the MTT results obtained for these drugs (5-FU and FdU) applied to the two colorectal cancer cell lines (Figure S4), we observed that both types of cells are resistant to these two anti-neoplastic drugs with a slightly higher antiproliferative effect produced by FdU in both cells lines and it is also possible to see a smaller increase on the cytotoxic effect in HCC2998 compared with HTB38. In contrast, in HeLa cells, FdU presented a higher antiproliferative effect even when it is compared to FU (Figure S4).

**Figure 3:**
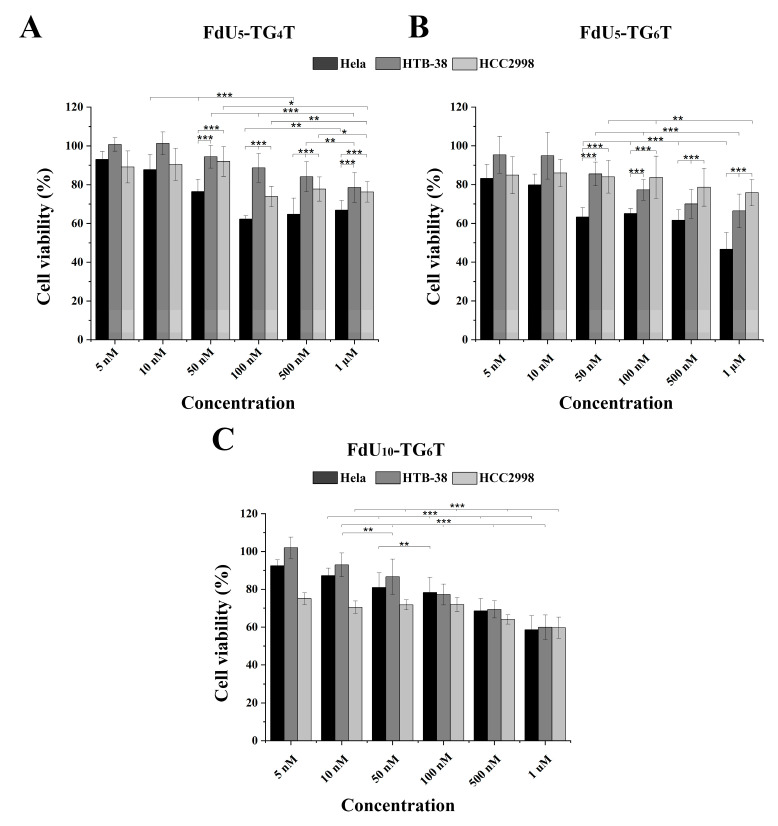
MTT cytotoxicity assay for the three DNA G-quadruplexes nanostructures in the HeLa (black), HTB-38 (dark grey) and HCC2998 (light grey). Cells were treated with (FdU)_5_-TG_4_T (panel (**A**)) and (FdU)_5_-TG_6_T (panel (**B**)) FdU_10_-TG_6_T (panel (**C**)) at a range of concentrations from 5 nM to 1 µM in panel A and B and 10 nM to 1 µM in panel C. Error bars represent the standard deviation (SD) of three independent experiments in triplicate (n = 9). * corresponds to t-test results with * *p* ≤ 0.1, ** *p* ≤ 0.05, *** *p* ≤ 0.01 significantly different within each indicated pair.

The cytotoxic effect observed in the three cancer cell lines by (FdU)_5_-TG_4_T, (FdU)_5_-TG_6_T and for 5-FdU-T_8_ was studied. Figure 3A,B show the cytotoxicity induced by (FdU)_5_-TG_4_T and (FdU)_5_-TG_6_T respectively. For (FdU)_5_-T_8_ a slight cytotoxicity is detected by MTT in the three cell lines (data not shown). The two FdU_5_ G-quadruplexes have similar cytotoxicity in HCC2998 (Figure 3A,B) reducing the value of cell viability by 25%, similar to the effect produce from FU and FdU (Figure S4). In the case of HTB38, a different behavior is observed for the two nanostructures. While (FdU)_5_-TG_4_T reproduces the same or even slightly lower effect (22%) as found in HCC2998, the (FdU)_5_-TG_6_T increase cell dead up to 30%. This cytotoxic effect is significantly higher than the one produced by 5-FU and FdU. These differences in the antiproliferative activity of the FdU_5_-TGxT quadruplexes is highly accentuated in HeLa cells where (FdU)_5_-TG_6_T achieves its IC_50_ value around 1 µM concentration. This is a similar value to the one observed for FdU but significantly higher than 5-FU. Even so, (FdU)_5_-TG_4_T produces a higher cytotoxic effect being around 40% in HeLa cells compared with the two colorectal cell lines.

Considering the promising result obtained with TG_6_T as nanocarrier in both colorectal cancer lines a new nanostructure with 10 units of FdU instead of 5 units with this carrier was evaluated as cytotoxic agent. Surprisingly, this new construct does not increase its killing effect in HeLa cells. However, in the two colorectal cells, we observed an increase in cell death of 20% HCC2998 and 10% HTB38 (Figure 3C). These results confirmed that the increase in the concentration of FdU per nanostructure increases the cytotoxicity in the more resistant cells reaching a similar cytoxocity that in HeLa cells.

**Figure 4:**
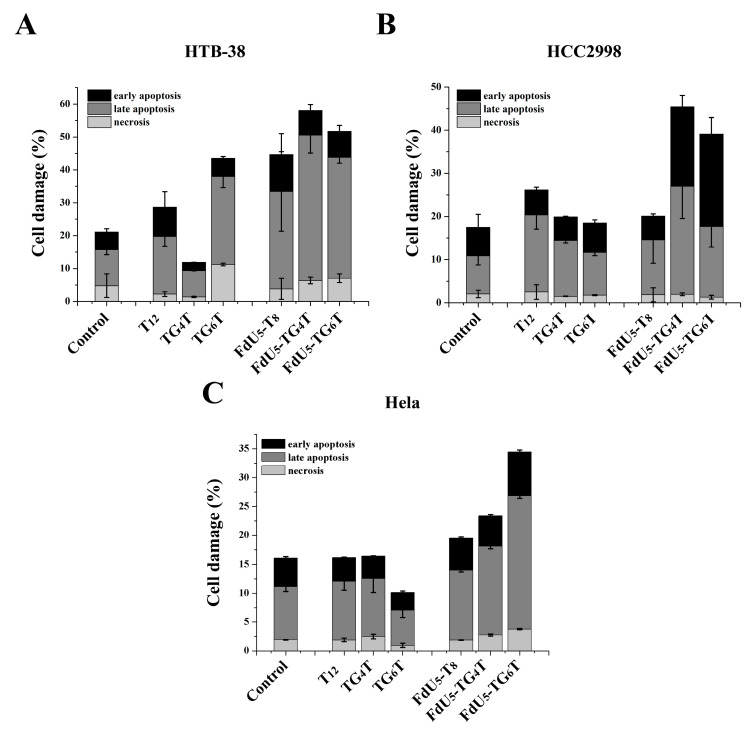
Apoptosis assay comparing TG_4_T and TG_6_T along or carrying 5 molecules of FdU, in (**A**), HeLa (**B**), HTB-38 and (**C**), HCC2998. Error bars represent the standard deviation (SD) of two independent experiments in duplicate.

Apoptosis assays were conducted to compare (FdU_5_)-TG_4_T and (FdU_5_)-TG_6_T effects on cell death and identify the cellular death mechanism involved in the reduction of cell viability. HCC2998 cells showed similar percentages of cell apoptosis and necrosis than HeLa (Figure 4). Unexpected by previous assays, HTB38 presented a higher level of apoptosis when compared to HCC2998 and HeLa cells. Based on the data shown in Figure 4, TG_4_T and TG_6_T increased cell damage in all cell lines, presenting the higher effect in the colorectal HCC2998 compared with T_8_ when FdU is incorporated indicating that the formation of the G-quadruplexes leads to an increase in the internalization ability to promote cell damage and that the resistance to FdU can be overcome with the help of a nanocarrier.

In comparison, the formation of an extra tetrad in TG_6_T favors cell damage in HeLa cells producing a distinguished behavior between the two G-quadruplexes as also shown in MTT assay. HCC2998 cells, conversely, do not present a difference in the actions of the two G-quadruplexes, even TG_4_T with an increase in later apoptosis performed slightly better also in this cell line we could observe an increase in the early apoptosis event.

## 3. Discussion

Antiproliferative nucleosides such as floxuridine, gemcitabine, cytarabine have been the drug of choice for certain malignancies during the last 40 years. Their mechanism of action implies the generation of the nucleoside 5′-monophosphate and combines the inhibition of enzymes involved in nucleic acids metabolism and the conversion of the nucleoside triphosphate followed by misincorporation into DNA and RNA generating DNA damage products, such as double-strand breaks, that induce apoptosis. Unfortunately, after some time of treatment it appears drug resistance mechanisms that prevent the formation of the active drug such as nucleoside 5′-phosphate derivates as well as toxicity issues coming from the nucleosides or their metabolites.

Some time ago, an innovative approach was described to improve the activity and reduce the toxicity issues [63]. This approach uses oligonucleotides composed of several units of antiproliferative nucleosides that are activated by the degradation of the oligomers by nucleases generating the active nucleoside 5′-monophosphates [64]. Decafloxuridine is one of the most studied antiproliferative oligomers showing a potent antitumoral activity in leukemias, glioblastoma and colon cancer [74] by inhibition of thymidylate synthase and DNA topoisomerase [75,76] with lower toxicity than FU as the formation of the toxic α-fluoro-β-alanine metabolite [77] is highly reduced compared with the use of FU [78].

Recently the advent of nanomedicine with the precise preparation of nanoparticles has triggered a large expectation for the development of nanomaterials for the specific targeted delivery of highly active antitumoral drugs to target tumor cells avoiding potential toxic effects in healthy tissues [79]. In this area the use of antiproliferative oligomers is of special interest as nanoparticles carrying special targeting molecules can be easily functionalized with these oligomers ensuring the selective delivery of the well-known antimetabolites to the tumor cells. Several examples of this approach can be found in the bibliography such as the use of proteins nanoparticles carrying peptides with affinity to CXCR_4_ receptors present in metastasic cells [65], the use of DNA aptamers modified with gemcitabine [57], and the use of DNA nanostructures to deliver gemcitabine [67] and floxuridine [66] to tumor cells.

In spite of these excellent results with complex but highly defined nanoparticles, we aimed to prepare the minimal DNA nanostructure able to direct antiproliferative oligomers to tumor cells. Inspired by the good results obtained with the use of G-rich aptamers such as AS1411 [58,59] with high affinity to nucleolin and other G-quadruplex binding proteins present in tumor cells we aimed to study the potential use of a simple TG_n_T sequence. In this way, if successful, a single short oligonucleotide (11 or 13 mer) will be able to tetramerize to obtain a nanostructure with a substantial affinity for some cancer cells by membrane protein -G-quadruplex interactions.

To this end, we have prepared the appropriate pentafloxuridine TG_4_T and TG_6_T oligonucleotides and the corresponding control pentafloxuridine-T_8_ sequence. One of the first concerns was if the presence of floxuridine oligomer prevented the formation of the tetrameric parallel G-quadruplexes. This was easily ruled out by recording the CD spectra of the floxuridine oligonucleotides confirming the presence of the characteristic bands described for parallel quadruplexes except in the control pentafloxuridine-T_8_ oligonucleotide (Figure 1A). Moreover, the analysis of the native gel electrophoresis shows the retardation of the bands corresponding to the G-quadruplex-forming oligonucleotides compared with the polythymidine controls. This is consistent with the results found previously with antiviral parallel G-quadruplex such as Hotoda’s sequence [80,81]], lipoquads [60] and antisense against luciferase [61,62].

Using the corresponding fluorescein labelled oligonucleotides the relative cellular uptake efficiency was studied in three different cancer lines including human cervix epithelioid carcinoma HeLa cells and two FU-resistant colon cancer cells (HCC2998 and HTB38). Both parallel G-quadruplex oligonucleotides carrying floxuridine were rapidly taken up by cancer cell lines especially by HeLa cells that are known to have abundant nucleolin and other G-quadruplex binding proteins. On the other hand, T_8_ oligonucleotides with and without floxuridine are not internalized. Although floxuridine-T_8_ oligonucleotide has a small increase in cellular uptake compared to T_8_ alone, the difference between the G-quadruplex oligonucleotides and T_8_ oligonucleotides are very clear achieving more than 80% of the internalization in the best case (HeLa cells and TG_6_T-floxuridine).

As a consequence of the increase in internalization, the MTT cytotoxicity and apoptosis assays show an increase of the cytotoxicity for the G-quadruplex oligonucleotides functionalized with floxuridine and especially in HeLa cells. Altogether we have demonstrated that the simple addition of six (TG_4_T) or eight (TG_6_T) residues to the antiproliferative oligomers can generate an increase efficacy by tetramerization of these well-known antitumoral drugs probably due to an increment in the internalization to cancer cells due to the presence of receptor proteins with affinity to G-quadruplex (see Figure S5). This strategy can be extended to other receptor-mediated strategies as well as extrapolated to antiviral nucleosides for increasing their biomedical properties. Moreover, the use of monomolecular quadruplexes [40,58,59] is an interesting option that we would like to explore in the near future. 

## 4. Materials and Methods

### 4.1. Synthesis of the Oligonucleotide Sequences

All the oligonucleotide sequences used in this study (see Table 1) were synthesized on an ABI 3400 DNA Synthesizer (Applied Biosystems, Foster City, CA, USA), in several batches of 1 µmol scale (CPG) synthesis and using the standard DMT-off protocols [82]. Fluoresceine (FAM) and FdU phosphoramidites were site-specifically inserted at the 5′-end of all the sequences.

The oligonucleotides were deprotected with ammonia solution overnight at 55 °C. Then, the ammonia solutions were concentrated to dryness and the residue was desalted by using a NAP-10 column (GE Healthcare (Little Chalfont, UK). The length and homogeneity of the oligonucleotides were checked by MALDI-TOF (Applied Biosystems, Foster City, CA. USA), Table 1). The modified oligonucleotide concentration was determined by absorbance measurements (260 nm) and their extinction coefficient calculated. All these samples were kept dry at −20 °C until further use.

### 4.2. G-quadruplex Formation

All the oligonucleotides conjugates were dissolved in 1× phosphate-buffered saline (PBS) pH 7.4. Then, to form the G-quadruplex nanostructures these solutions were annealed by heating at 94 °C for 2 min and slowly cooled down to room temperature for a week. The resulting G-quadruplex oligonucleotide conjugates and the control sequences were stored at −20 °C. PBS is phosphate buffered saline containing disodium hydrogen phosphate, sodium chloride and potassium chloride.

### 4.3. Circular Dichroism

Circular dichroism (CD) spectra of the G-quadruplex conjugates, the unmodified G-quadruplexes and the control sequences were recorded at the same concentration and using PBS (pH 7.4) as a buffer (Jasco V650 spectrophotometer, Madrid, Spain). The spectra were registered at 25 °C, over a range of 205–320 nm with a scanning speed of 100 nm/min, a response time of 4 s, a 0.5-nm data pitch, and a 1-nm bandwidth. 

### 4.4. Native Electrophoretic Mobility Shift Assay

G-quadruplexes (0.5 µM) were analyzed by electrophoresis on 20% polyacrylamide gel under native conditions. 1× TBE (supplemented with 100 mM KCl) was used as a running buffer. The corresponding tetramer was dissolved in a mixture containing 1x TBE (supplemented with 100 mM KCl) and 50% glycerol (1:1) and the sample was run at 150 V for at least 3 h at 20 °C. All bands were stained with Stains-All (Sigma-Aldrich, Madrid, Spain) according to the manufacturer’s instructions. Unmodified oligonucleotides TG_n_T and T_n_ were used as controls.

### 4.5. Internalization by Flow-Cytometry

HTB-38 and HeLa cells lines (HT-29 (ATCC^®^ HTB-38) and HeLa (ATCC^®^ CCL-2)), were purchased from the American Type Culture Collection (Manassas, VA, USA) and the HCC2998 cell line was kindly provided by Dr. Diego Arango (Molecular Oncology Group; CIBBIM-Nanomedicine, Vall d’Hebron Institut of Research (VHIR, Barcelona, Spain). To assess the level of internalization of DNA nanostructures modified with FdU_5_ the following procedure was conducted. Cells were seeded in 24-well plates at a density of 8 × 10^4^ cell well^−1^ and incubated for 24 h prior experiment. The tested DNA nanostructures were added directly to the cells dissolved in fresh growth medium and incubated. Twenty-four hours after samples addition, cells were washed once with 1 × PBS and harvested by treatment with trypsin, taken up in cell culture medium and centrifuged at 1000 rpm for 8 min. To evaluate the possible fluorescence due to the membrane-bound fluorescein, the cells were resuspended in 87.2 uL of PBS and treated with 12.5 uL of a solution of trypan blue 0.2% for 1 min and then washed with PBS and centrifuged at 1000 rpm for 8 min. The remaining supernatant was suspended in cold PBS and propidium iodide (PI) was added to stain dead cells. For about 5000 events were recorded for every experimental condition and each experiment was conducted in duplicate.

### 4.6. Apoptosis by Flow-Cytometry

The proportion of apoptotic cells resulting from exposition to the G-quadruplex nanostructures modified with FdU_5_ was evaluated by flow cytometry combining fluorescein isothiocyanate (FITC)-Annexin V and PI. Cells were seeded in duplicate in 24-well plates with a density of 5 × 10^4^ cell/well. Cells were treated with the FdU_5_-nanostructures and the controls and incubated for 48 hours. The attached cells were harvested with trypsin and added to floating cells all of them were centrifuged at 1000 rpm for 8 min. Next, the samples were washed once in cold PBS and centrifuged again. The final pellets were resuspended in Annexin binding buffer plus FITC-Annexin V and PI according to the manufacturer’s specifications. After 15 min of incubation, the samples were analyzed by flow cytometry (Millipore, Burlington, MA, US). As well, 5000 events were recorded for every experimental condition and each experiment was conducted in duplicates.

### 4.7. Cell Proliferation Assay (MTT)

To determine the effect of the G-quadruplex nanostructures modified with FdU_5_ on cell proliferation, the method of 1-(4,5-dimethylthiazol-2-yl)-3,5-diphenylformazan (MTT) reduction was carried out. Three cell lines HTB-38, HCC2998 and HeLa were seeded at 4–5 × 10^4^ cell/well in a 96-well plate after ensuring that the seeding density was suitable to guarantee 80% confluence during experiment completion. Twenty-four hours after plating, samples dissolved in sterile saline buffer were added in the medium to the cells as well as the respective negative controls. FdU were dissolved in sterile 0.5% (*v*/*v*) DMSO in culture medium and plated in the same range of concentrations used for the DNA oligonucleotides sequences. After 48 hours of incubation at 37 °C under 5% CO_2,_ the MTT reagent was added to a final concentration of 0.7 mg mL^−1^ to each well and incubated for 2 h. The MTT reduction was read at a single wavelength of 570 nm. Each experiment was conducted at least in triplicate (n > 3).

### 4.8. Statistical Analysis

The statistical significance of replicates was analyzed using the Student’s t-test with * *p* ≤ 0.1, ** *p* ≤ 0.05, *** *p* ≤ 0.01.

## 5. Conclusions

In this work, we investigated the effect of the presence of a parallel G-quadruplex in the antiproliferative activity of pentafloxuridine oligomers to several human cancer cells. First, we demonstrated that the presence of (FdU)_5_ at the 5’-termini of these two nanostructures combining 4 and 6 tetrads did not disrupt the formation of the expected parallel G-quadruplex. Interestingly, the presence of the G-quadruplex facilitated the internalization of the FdU oligomers increasing the cytotoxic properties of FdU oligomers and the increase in the number of FdU attach to the same nanocarrier is advantageous in the case of the two colorectal cancer lines with are more FdU resistance. All together we can conclude that the introduction of both TG_4_T or TG_6_T sequences to oligomers of antimetabolite nucleosides such as floxuridine has a clear benefit for the antitumoral activity of these oligomers that most probably comes from the facilitation of cellular uptake mediated by the interaction of G-quadruplex with protein receptors present in cancer cells as these carriers are not cytotoxic per se.

## Figures and Tables

**Table 1:** Sequence and mass spectra of the DNA- oligonucleotides.

Name	Sequence (5′-3′)	MW Calc	MW Exp
TG_4_T	TGGGGT	1861.3	1860.0
TG_6_T	TGGGGGGT	2519.4	2517.1
T_8_	TTTTTTTT	2369.4	2364.4
FdU_5_-T_8_	FdU-FdU-FdU-FdU-FdU-TTTTTTTT	3909.5	3912.5
^1^ FdU_5_-TG_4_T	FdU-FdU-FdU-FdU-FdU-TGGGGT	3401.4	3399.2
FdU_5_-TG_6_T	FdU-FdU-FdU-FdU-FdU-TGGGGGGT	4059.5	4063.8
FdU_10_-TG_6_T	(FdU)_10_-TGGGGGGT	5599.6	5601.4
^2^ FAM-TG_4_T	FAM-TGGGGT	2399.5	^3^ n.d.
FAM-TG_6_T	FAM-TGGGGGGT	3057.57	3053.9
FAM-T_8_	FAM-TTTTTTTT	2907.6	2902.8
FAM-FdU_5_-T_8_	FAM-FdU-FdU-FdU-FdU-FdU-TTTTTTTT	4447.6	4447.7
FAM-FdU_5_-TG_4_T	FAM-FdU-FdU-FdU-FdU-FdU-TGGGGT	3971.6	3983.4
FAM-FdU_5_-TG_6_T	FAM-FdU-FdU-FdU-FdU-FdU-TGGGGGGT	4629.7	4685.1

^1^ FdU: 5-fluoro-2′-deoxyuridine, ^2^ FAM: fluorescein fluorophore, ^3^ n.d.: not determined.

## Data Availability

The data presented in this study are available on request from the corresponding author.

## Supplementary Materials

The following are available online, Materials and methods and biophysical data of unmodified TG_4_T and TG_6_T control oligonucleotides. Figure S1 raw data of the electrophoresis analysis. Figure S2 Intracellular uptake of G-quadruplex nanostructures controls. Figures S3 and S4 MTT cell viability assays of control oligonucleotides, 5-fluorouracil and 5-fluoro-2′-deoxyuridine. Figure S5 Schematic diagram of the mechanism of action.

## Sample Availability

Samples of the FdU oligonucleotides are available from the authors upon request to the corresponding author.
