# Supplementary material for: Parallel G-quadruplex Structures Increase Cellular Uptake and Cytotoxicity of 5-Fluoro-2′-deoxyuridine Oligomers in 5-Fluorouracil Resistant Cells
Source: Molecules. 2021 Mar 20;26(6):1741. doi: 10.3390/molecules26061741 (PMC8003610; doi:10.3390/molecules26061741)
Supplement: Supplementary file 1 [file molecules-26-01741-s001.pdf]

Supporting Information

Parallel G-quadruplex structures increase cellular uptake and cytotoxicity of 5-fluoro-2′-deoxyuridine oligomers in 5-fluorouracil resistant cells.

Anna Clua[1], Carme Fàbrega[1], Jesús García-Chica[1], Santiago Grijalvo[1], and Ramon Eritja[1,*]

Institute for Advanced Chemistry of Catalonia (IQAC-CSIC), Networking Center on Bioengineering, Biomaterials and Nanomedicine (CIBER-BBN), Jordi Girona 18-26, E-08034 Barcelona, Spain. anna.clua@iqac.csic.es (A.C.); cgcnqb@cid.csic.es (C.F.); garciachicajesus@gmail.com (J.G.C.); sgrgma@cid.csic.es (S.G.)

Corresponding Author

* Email; recgma@cid.csic.es (R. E).

Table of contents:

1. Materials and methods and biophysical data of unmodified TG_4T and TG_6T control oligonucleotides — Page 2
2. Figure S1: Raw data from the 20% polyacrylamide gel electrophoresis analysis of the FdU conjugates — Page 4
3. Figure S2. Intracellular uptake of G-quadruplex nanostructures controls — Page 5
4. Figure S3: Cell viability assay of G-quadruplexes controls sequences — Page 6
5. Figure S4: MTT cell viability assay of 5-FU and FdU — Page 7
6. Figure S5: Schematic diagram of the mechanism of action — Page 8

Material and methods

Reagents: The standard phosphoramidites and ancillary reagents used on the oligonucleotide synthesis were obtained from Applied Biosystems (Foster City, CA, USA) and LGC Link (Lanarkshire, Scotland, UK). 5′-Fluorescein CE phosphoramidite (FAM), and 5-FdU-CE phosphoramidite were acquired from LGC Link (Lanarkshire, Scotland, UK). 5-Fluoro-2′-deoxyuridine (FdU) was purchased from Alfa Aesar (Thermo Fisher, Kandel, Germany). Matrix for MALDI-TOF experiments was composed by 2′,4′,6′-trihydroxiacetophenone monohydrate (THAP, Sigma-Aldrich, Madrid, Spain) and ammonium citrate dibasic (Fluka, Sigma-Aldrich, Madrid, Spain). The desalting columns with Sephadex G-25 (NAP-10 were from GE Healthcare (Little Chalfont, UK). The rest of the chemicals are analytical reagent grade from commercial sources as specified. Ultrapure water (filtered through Millipore filters) was used in all experiments. HTB-38 and HeLa cells lines were purchased from American Type Culture Collection (HT-29 (ATCC® HTB-38) and HeLa (ATCC® CCL-2)), Manassas, VA, USA and HCC2998 cell line was kindly provided by Dr. Diego Arango (Molecular Oncology Group; CIBBIM-Nanomedicine, Vall d'Hebron Institut of Research (VHIR)). Cell lines were cultured in Nunc® 10cm culture dishes (Thermo Scientific™) (Waltham, MA, USA) and were grown in DMEM supplemented with 10% fetal bovine serum (FBS). Cultures were grown at 37 °C in 5% CO_2 humidified atmosphere. DMEM (Dulbecco's Modified Eagle Medium), PBS (phosphate buffered saline containing disodium hydrogen phosphate, sodium chloride and potassium chloride) and RNAse free water were purchased from Gibco (Waltham, Massachusetts, USA). Annexin V-FITC kit was obtained from ThermoFisher and used following manufacturer's instructions.

Instrumentation: Modified oligonucleotides were synthesized on an ABI 3400 DNA Synthesizer (Applied Biosystems, Foster City, CA. USA). Mass spectra were recorded on a MALDI Voyager DE™ RP time-of-flight (TOF) spectrometer (Applied Biosystems, Foster City, CA. USA). Molecular absorption spectra between 220 and 550 nm were recorded with a Jasco (Madrid, Spain) V650 spectrophotometer. The temperature was controlled with an 89090A Agilent (Santa Clara, CA, USA) Peltier device. Hellma (Jena, Germany) quartz cuvettes were used. Gels were imaged with a Gene Genius Bioimaging system (Syngene International Ltd. Bangalore, India).

The MTT and colony assays were measured in an automated spectrophotometric plate reader Glomax multi detection system (Promega, Madison, WI, USA)). Internalization and apoptosis assays were measured with a Guava easyCyte™ flow cytometer (Millipore, Burlington, MA, USA) and data was analyzed with Guavasoft 3.1.1.

Biophysical data of unmodified TG_4T and TG_6T control oligonucleotides. CD spectra of TG_4T and TG_6T control oligonucleotides are described in the bibliography (Dapic et al. Nucleic Acids Res. 2003, 31, 2097- 2107, Grijalvo et al. RSC Adv., 2016, 6, 76099, Grijalvo et al. Int. J. Mol. Sci. 2021, 22, 121). Melting curves of the unmodified oligonucleotides can also be found in the bibliography. TG_4T melting can only be observed in sodium buffers but the melting temperatures of the six tetrads quadruplex (TG_6T derivatives) cannot be measured because they do not melt even at temperatures higher than 80ºC. For example TG_4T melts at 59.4ºC but TG_6T cannot be measured (>80ºC) because no melting can be observed up to 80ºC (in 10 mM sodium cacodylate buffer with 0.15 M NaCl (pH 7.2), Grijalvo et al. RSC Adv., 2016, 6, 76099, Grijalvo et al. Int. J. Mol. Sci. 2021, 22, 121). In another study (Ferreira et al. ChemistryOpen 2012, 1, 106-111) the effect of the salt on the melting of TG_4T gave the following melting temperatures: 58 ºC (100 mM NaCl); 67 ºC (100 mM NH_4OAc) and >80ºC (5 mM KCl). For the modified TG_4T and TG_6T oligonucleotides carrying an antisense oligonucleotide with lipids and positively charged ligands we cannot observe any melting behavior as G-quadruplex are stable up to 80ºC (Grijalvo et al. RSC Adv., 2016, 6, 76099, Grijalvo et al. Int. J. Mol. Sci. 2021, 22, 121).

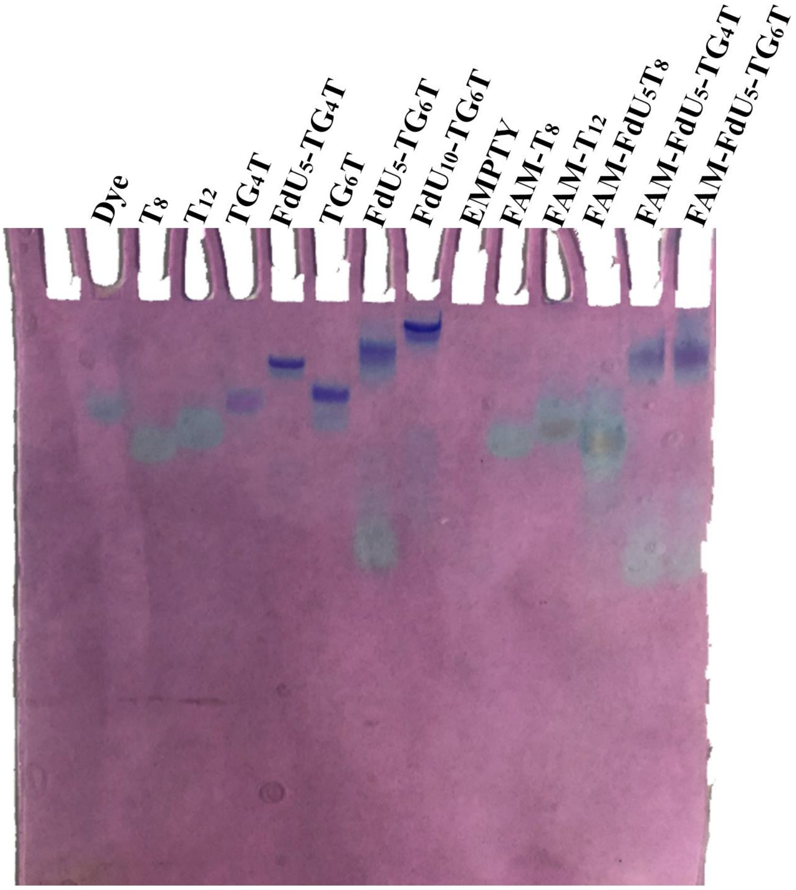

Figure S1: Raw data from the 20% polyacrylamide gel electrophoresis analysis of the FdU conjugates structures formed upon incubation of the different DNA sequences in TBE (tris-borate EDTA) buffer supplemented with 100 mM KCl

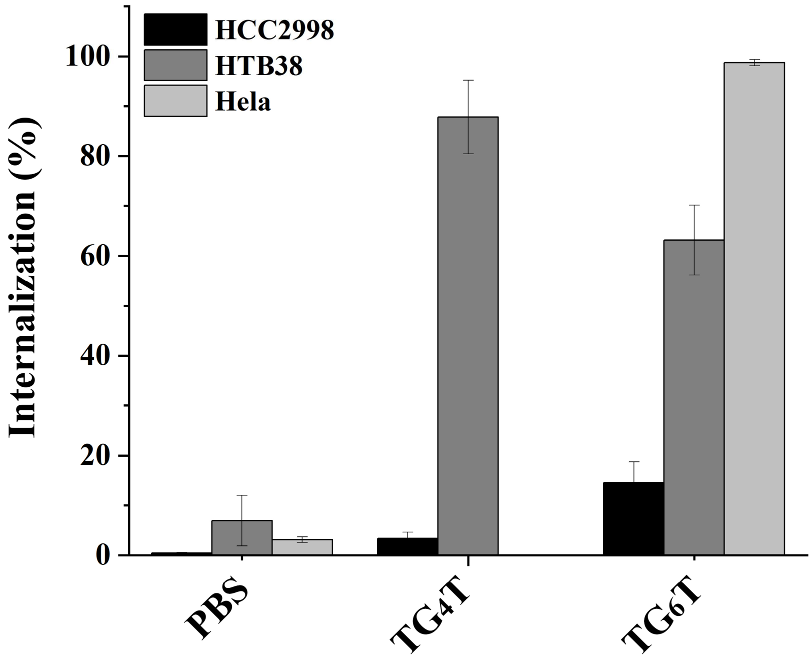

Figure S2. Intracellular uptake of G-quadruplex nanostructures controls in HeLa, and two colorectal cancer cells (HCC2998 and HTB38). Cells were incubated separately with the native and modified G-quadruplexes at 1 μM concentration, and the internalization is shown in the bar graphs HCC2998 (black), HTB38 (dark grey) and HeLa (light grey). Error bars represent the standard deviation (SD) of two independent experiments in duplicate.

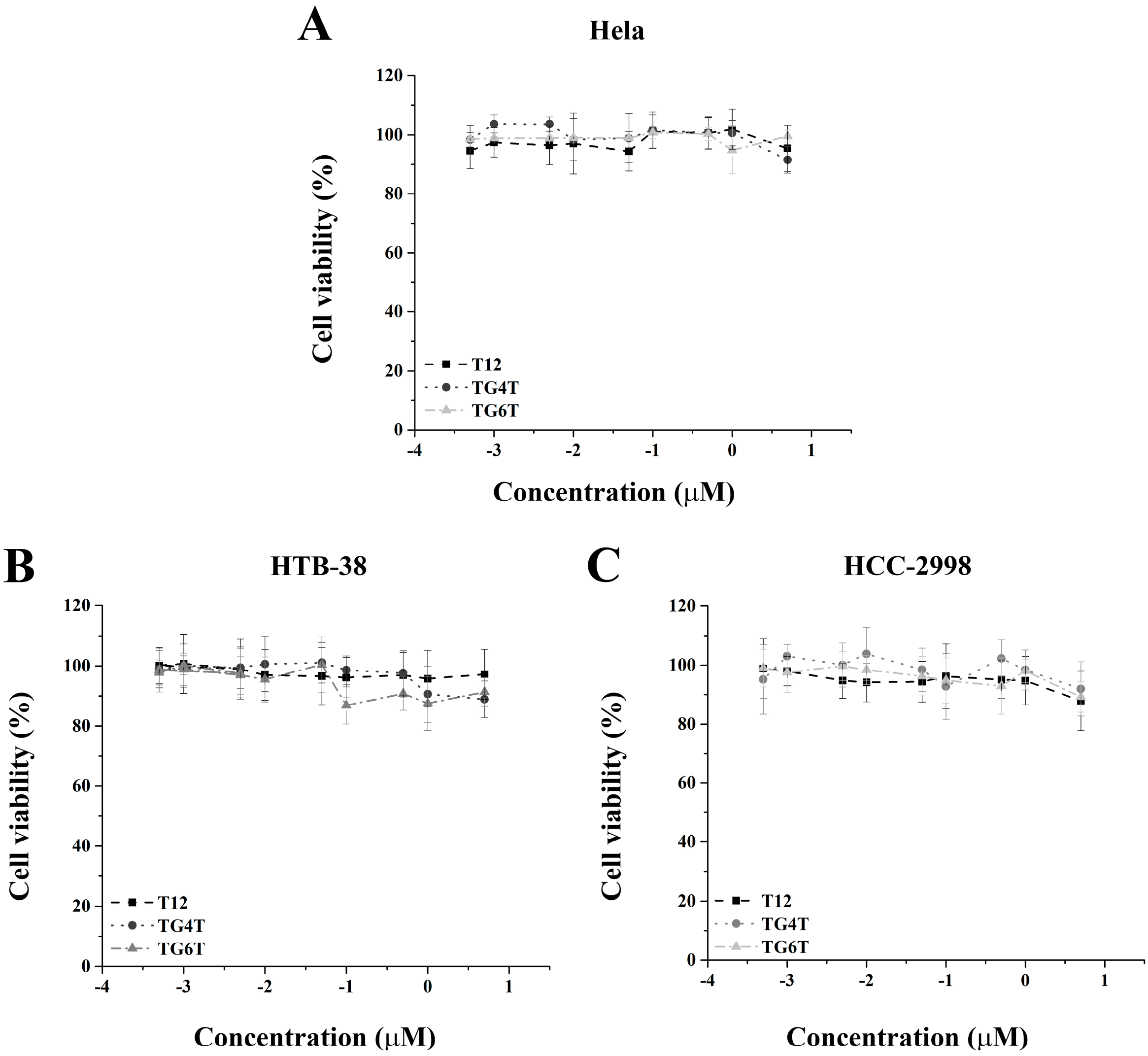

Figure S3: MTT cell viability assay. The two G-quadruplexes and the T_{12} control sequences were assayed in HeLa (A) and two different types of colorectal cancer cell lines HTB-38 (B) and HCC2998 (C). The values obtained for T_{12}, TG_4T and TG_6T are shown as fill square, cycles and triangles, respectively. All data are normalized to PBS used as the control, as it was used to dissolve the oligonucleotide sequences. The concentration assayed range from 10 nanomolar (nM) to 5 micromolar (μM). Each graphic represents the average of duplicates of three separate experiments.

The lack of toxicity of TG_4T and TG_6T is in agreement with previous bibliographic data (Dapic et al. Nucleic Acids Res. 2003, 31, 2097- 2107, Grijalvo et al. RSC Adv., 2016, 6, 76099; Grijalvo et al. Int. J. Mol. Sci. 2021, 22, 121).

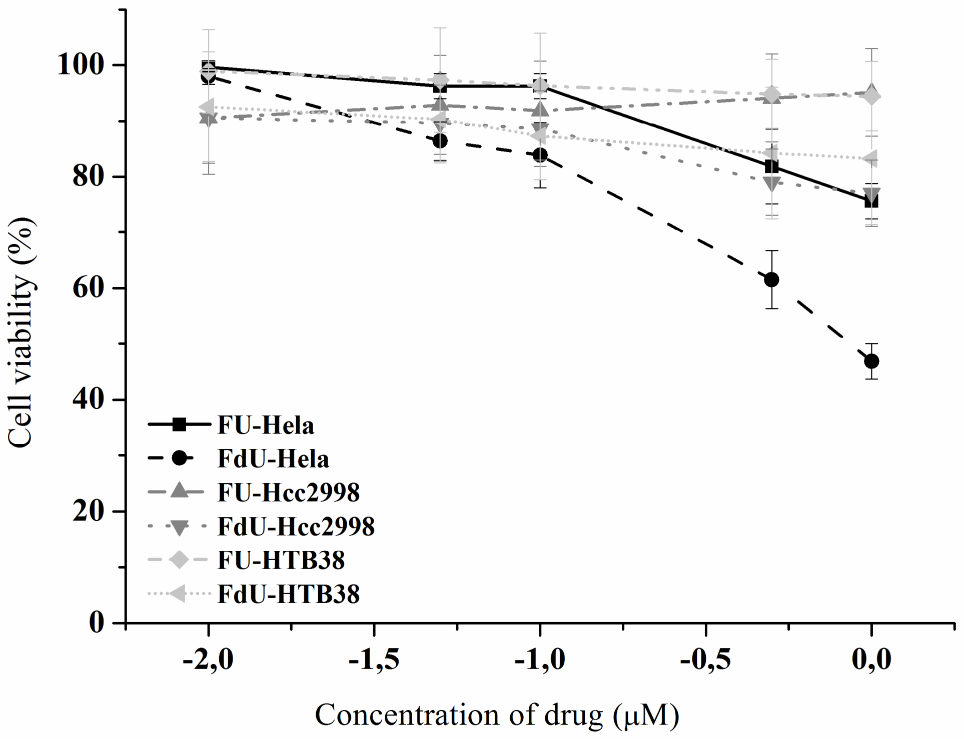

Figure S4: MTT cell viability assay. 5-fluorouracil (5-FU) and 5-fluoro-2′-deoxyuridine (FdU) were assayed in two different types of colorectal cancer cell HTB-38, HCC2998 and in HeLa. All data are normalized to DMSO used as a control, as it was used to dissolve the two drugs. The concentration assayed range from 10 nanomolar (nM) to 1 micromolar (μM). Each graphic represents the average of duplicates of three separate experiments.

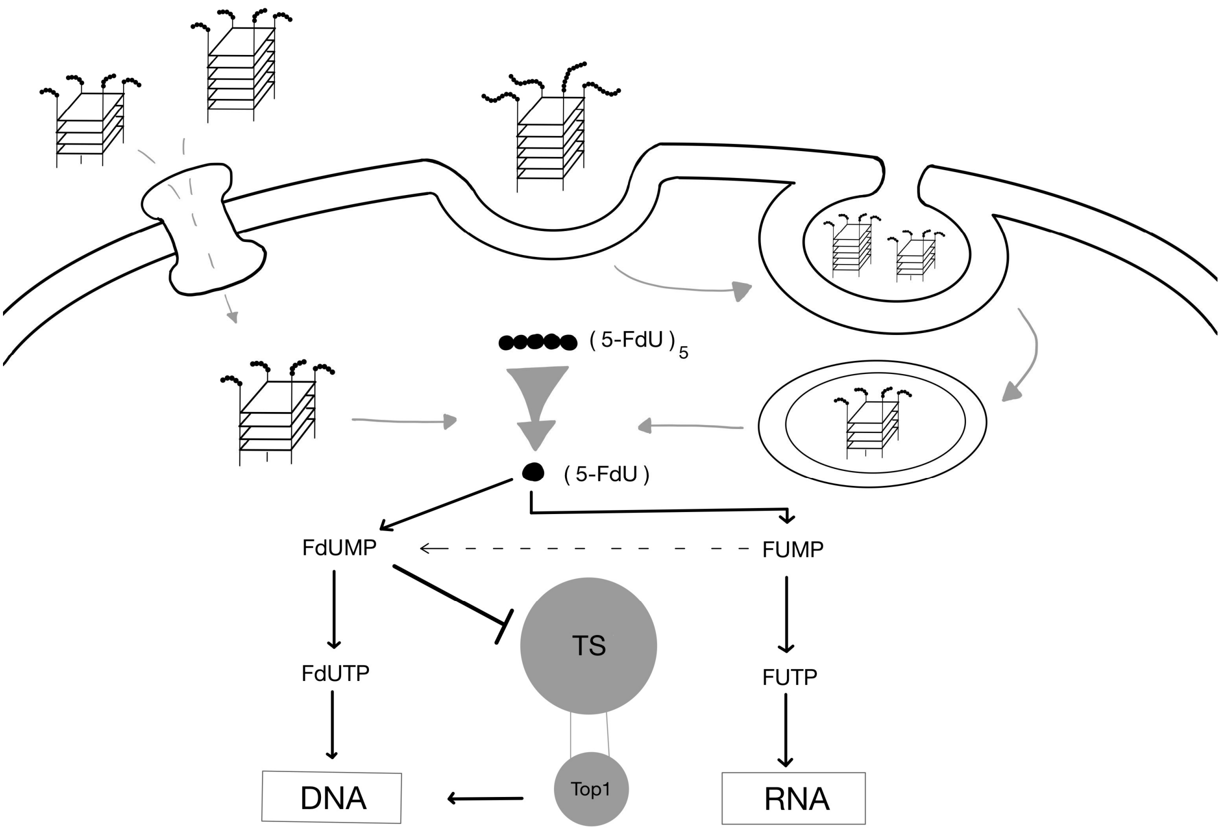

Figure S5: Schematic diagram showing the potential mechanism of action of the antiproliferative properties of the G-quadruplexes functionalized with FdU oligomers.
